# Diseased Gums

**Published:** 1877-07

**Authors:** L. C. Ingersoll

**Affiliations:** Keokuk, Iowa.


AMERICAN JOURNAL

OF

DENTAL SCIENCE.

Vol. XI. THIRD SERIES—JULY, 1877. No. 3.

### ARTICLE I.

### *Diseased Gums.*

AN ESSAY BY L. C. INGERSOLL, KEOKUK, IOWA.

Read before the Iowa State Dental Society, May 2nd, 1877.

It is my wish in this paper to call attention to a subject which, it seems to me, is too often neglected by the profession. It is not my purpose, however, to give a thorough presentation of the varied diseased conditions to which the gums are subject ; but to notice those forms of disease most commonly met with and too often neglected. No mouth can be considered in a condition of health, however sound the teeth, unless the gum surrounding the teeth is healthy, and no dentist, in any treatment of the teeth, should consider them in a safe condition until the gums are restored to healthful firmness and color.

It is true of mankind generally that morbid and diseased conditions are not recognized unless attended with suffering, and usually, suffering of an acute character. Local disease of the gums is, in most cases, of such slow development as to be unnoticed, and the increase of the disease is so gradual as to arouse no suspicion of disastrous consequences. It becomes the duty of the dentist, therefore, to observe and to inform his patient of the threatening danger. The prevalence of diseased gums even in the mouths of those who are accustomed to employ dental skill on their teeth, shows neglect, or failure in treatment, on the part of the profession.

The evil of diseased gums is apparent in several particulars. Their thickened condition induced by inflammation, affords a lodgment for food and viscid mucus, which renders the mouth unsightly. The gums being tender to the touch, this foreign matter is not thoroughly removed. What remains, soon undergoes decomposition and emits a foul odor to taint the breath. The fermenting fluid in contact with the gum, irritates and increases the inflammation, till suppuration is induced or fungous growths appear, and disgusting tumors void their waste disorganized matter into the mouth. This vitiated fluid mingling with the saliva and food passes daily into the stomach to continue its harmful effect there. And we might trace it still farther, in a series of inevitable consequences that bring misery to life and terminate, in some instances, in death.

I have distinctly in mind now a case in my early practice, in which diseased gums, progressing slowly for a period of fifteen years, had deprived the stomach of its power, had undermined the nervous system, had seriously affected the bronchial tubes, and the patient had wasted away to a mere skeleton, confined to the house, and obliged daily to resort to her bed to recuperate the strength necessary to walk feebly about her room. All this while she was under the care of a physician of education, skill and experience who had bolstered her up with anodynes and tonics, but who never conceived the cause of her complicated troubles. Pain was the habitual experience of her life. Mastication had been impossible for years. She ate, drank and breathed *pus*. Every tooth and root was loose, and not one could be touched without causing severe pain. I removed more than twenty teeth and fragments of teeth as the first step in the treatment—the most excruciating operation that I had ever before or have ever since performed. In a few days she was able to say to me, when I called upon her, "I am very weak, but I am free from pain, and *perfectly happy!*—which I could not have said during the past fifteen years."

Although the stomach, in regaining its tone, was the source of some trouble, relieving the gums of the irritating and painful presence of the teeth was the beginning of new life and hope for the woman. After treatment of her gums for a few weeks I did not see her again for a year, when I could not recognize my former patient. She had added one-third to her weight, and her countenance and walk were the expression of health—and ever since—now more than ten years—she has been a healthy woman.

This was an extreme case: not in the *nature* of the disease but in its extent—involving the entire gum of both jaws—and in its long continuance. Like conditions may be found in many mouths confined to small areas, but demanding the careful attention of the dentist.

To understand more fully the diseases of the gums we need to consider the structure of this tissue. Time will not permit a full description, but I will remind you of a few facts. The nerves of the gum except when diseased, are not very sensitive. Hence they will suffer great abuse without giving the warning of pain. But when diseased, they become peculiarly sensitive, and are disturbers of the general health not only through their nerve connections, but through the stomach, by rendering mastication impossible without pain, and thus over-taxing and weakening the whole digestive system.

The mucous membrane and the capillary vessels of a healthy gum are exceedingly firm and tough. But when these vessels have for a long time been distended by congestion, their coatings become thin and weak, and will burst on very slight touch, so that ordinary mastication tinges the saliva and the food with blood.

In this outer covering of the gum are located the mucous follicles wherein is secreted the mucus and from which it is poured forth to lubricate the gums and prevent abrasion from the friction of mastication. They are larger and more abundant in the mucous membrane covering the gum than in other parts of the mouth, for the reason that the lubricating effect of mucus is not needed there, and at the gingival margin they are particularly prominent in the form papillæ, giving it a fringed appearance, and they supply mucus abundantly. The mucus is a secretion from the watery portion of the blood, combined with the animal matter of the wasting tissue itself; so the mucous follicles are in a measure excretory ducts. In a diseased condition of the mucous membrane, the follicular secretions are so changed in their character as to become putrid almost immediately after exposure, and at the margin of the gum where the greatest irritation is produced this degenerate fluid becomes so acrid as to dissolve enamel. This, to my mind, is sufficient to account for the wasting of tooth substance in pitted and grooved horizontal lines across the crowns of teeth. These grooves and pits may sometimes be found midway from the margin of the gum to the grinding face of the molars or cutting edges of the anterior teeth; or the effect may be seen in the cusps of the molars and canines. For, each of these points of attack was once at the margin of the gum bathed by this acrid fluid. The appearance in each case is that of having been dissolved by some agent acting from without, and conforms to no law of "*suspended development*" during the process of tooth formation.

We see the same effect of this mucous decay of the teeth at every period in life, in lines of decay at the margins of the gum. But while the teeth are advancing by successive stages of development—growth and cessation alternating—there is time only for the formation of very shallow grooves which mark these alternating periods.

Beside this peculiarly organized mucous membrane, there is also, covering the gum, that indefinitely defined coating known as the *epithelium.* Some writers seem to confound this with the mucous membrane itself. By some it is considered as not having an organic structure at all, but having only a mechanical arrangement of the scales of which it is composed—and that these scales are only coagulated mucus. I shall not stop to consider whether it is vital or non-vital, but only refer to its connection with the mucous membrane.

The term *epithelium* is derived from two greek words *epi*, upon, and *thele*, a nipple. It is applied to designate a covering of some exposed parts of the body not covered by a true skin. While it is dermatoid in the purposes which it serves, it is not of the organic structure of the *cutis vera.* It is irregular in its thickness, scarcely perceptible around the papillæ of the mucous follicles from which it is continually rubbed off, while between them it is of such thickness as to be sometimes readily observed by the naked eye, giving a whitish appearance to the gum. Although it is sometimes spoken of as that in which the tooth germs have their origin, I have never seen any description of disease pertaining to the epithelium, to which, if it be an organic structure it must be liable.

Below the mucous membrane lies a thick firm tissue composing the body of the gum. In its outer part it is stellate and areolar in its structure, but growing more dense and fibrous in its inner portion where it unites with the periosteum of the alveolar border. It is well supplied with blood-vessels—most abundantly in its inner part. In this tissue is the chief seat of diseased gums. When oral disease pertains to the mucous membrane it will manifest itself in other parts of the mouth as well as on the gums—will be found in the fauces, and may extend down the esophagus to the cardiac opening into the stomach. This disease though found on the mucous membrane covering the gums could not be strictly denominated a *disease of the gums.*

So multitudinous are the causes, primary and secondary, predisposing and exciting, which produce disease of the gums, that I shall not even attempt an enumeration of them ; but shall allude only to such as are most common and plainly recognized and which demand immediate removal as a first step in the remedial treatment.

We are all too well aware of the condition of the tissues of the mouth arising from filthy habits. Not that dentists need special commiseration on this account. For, those who employ the services of a dentist—except it be for the momentary operation of extraction—are, for the most part, those who take care of their teeth and wish to preserve them. Particles of food, coagulated mucus and loosened epithelium scales tending to decomposition, cannot remain day after day in contact with the necks of the teeth without causing irritation of the gum and increase of vascular action. The gums are reddened and swollen.

In such cases the only treatment needed is home-treatment with toothpick, soap, and brush, daily and habitually applied. Gums unused to the brush are tender to its touch, and multitudes of persons, through the influence of newspaper advertisements of various dentifrices and tooth washes, are led to believe that they need some medicated powder or lotion to "harden their gums." While the fact is that in ninety-nine cases in a hundred, cleanliness and the friction of the brush persisted in, will, in a few days or weeks, give to the gums health and firmness. To one not habituated to performing this cleansing, a hand-glass is as needful as eyes are in doing any thing else to which we are not accustomed.

But, if in addition to this soft creamy accumulation mentioned above, tartar is found deposited, it needs the careful attention of the dentist, as no dentifrices or home treatment will remove it. Often this concretionary deposit will constitute the chief amount of the accumulation, varying in thickness according to location and form of the teeth. But the gums may not be so seriously affected by its irritating presence but that a careful and thorough removal of the deposit with instruments, followed by daily toilet cleansing, will effect a cure.

Not so, however, in every case. The tartar produces a congested, hypertophied and sensitive condition of the gum which will not permit the free use of the brush. The gum is loosened from the teeth, and the excessive growth of the gum in the inter-spaces of the teeth forms long points of gum extending from one-half to two-thirds the length of the crowns, behind which the deposit is continued, to remain a perpetual source of disease in spite of toilet cleansing. The gums must be reduced. How shall this be done?

It has been recommended in such cases, to scarify the gum freely and use astringent washes. The objection to this treatment is, that it requires too long a time, and lacks thoroughness.

Nitrate of silver has been recommended both for its astringent and caustic effect. I object to nitrate of silver because the nitric acid in the remedy, acts upon the tooth substance to destroy it; and the action of light upon the nitrate of silver blackens the teeth. Other caustics have been used with better effect and less harm to contiguous parts. But all caustics, continued in the treatment day after day, keep the mouth sore and deprive the patient of a comfortable meal during a long course of treatment.

My method is to remove with scissors the apices of the gum in the inter-spaces of the teeth and cauterize the whole gingival margin with carbolic acid. A single operation of this kind is in very many cases sufficient, if the parts are kept clean. Other cases may require the application of a caustic a second or a third time at intervals of two or three days. This method is speedy and thorough, and rarely attended with much pain. In worse cases, involving the entire margin of the gum, I have removed with knife and scissors the entire marginal border in one strip from the second bicuspid on the left around to the corresponding tooth on the right, and have witnessed the most decided beneficial results in two or three days time.

I prefer carbolic acid to other caustics in common use, because it has more power over morbid growths, and tends to promote healthy granulation over raw surfaces. Chloride of zinc is not so superficial in its action is less easily limited in its effects, and endangers the destruction of parts beyond what you desire, besides being very painful when applied. If only a slight caustic effect and stimulation is desired I would use creosote. Not the creosote commonly sold in the shops, made from coal tar, which scarcely differs from carbolic acid. But the old fashioned creosote made from pine tar, with the lingering odor of pitch in it.

This was formerly known as pyroligneous acid, and sometimes called oil of smoke. It is now quite difficult to obtain. It is more commonly the case that carbolic acid, and creosote are sold out of the same bottle. Because of this I desire here to notice some of their physical and chemical properties, by which they may be distinguished the one from the other. Creosote is an acetic acid combined in small proportion with various oily impurities. Carbolic acid is neither an acid, nor an oil, but is more nearly an alcohol. Creosote combines very sparingly with water, and only by agitation, while carbolic acid combines very readily and in all proportions with water. Creosote will not crystallize; carbolic acid in its pure and most caustic form readily crystallizes in all cool weather. Creosote changes color, and becomes very dark on exposure to light; carbolic acid is unchanged by light. Creosote is sparingly destructive to vital tissues, while carbolic acid is powerfully so. In all cases where it is desirable to produce a decided caustic effect, I prefer the crystalline carbolic acid. But for the removal of hypertrophied gum, nothing is so speedy, thorough and certain, as knife and scissors.

So far, I have alluded only to those cases of diseased gums affecting all, or a large number of the teeth, and caused by the presence of tartar or other foreign deposit. I wish now to notice those chronic conditions which, though caused by tartar, irritation of broken teeth, or other means, may exist in mouths well cared for, as a great source of annoyance to the patient, and endangering the loss of the teeth so affected.

Continual irritation may cause a tumefied condition of the gum. Sometimes limited to the apices of the gum in the inter-spaces of two or three teeth only. When examined with a glass, such diseased parts look like sponge or growing moss, and on very slight touch bleed freely, and are of the nature of the epulis tumor. In eradicating this disease, the operation must be thorough; cutting out fully to the line of the healthy gum, and through to the periosteum of the alveolus. Then bathe the cut surfaces with the caustic carbolic acid, to destroy any fibers of morbid growth that may possibly be left by the knife.

Perhaps the worst and most unmanageable of all troubles connected with the gums, is that wasting away of the gums and alveolar processes which first denuded the necks of the teeth, then the roots, till finally the loosening requires the extraction of the teeth as the only remedy. In some cases it is confined to a single tooth, to the anterior portion of an incisor or canine, or to the palatine root of an upper molar. In other cases several teeth are involved, as, for example, the inferior incisors; and the gum and parietes of the alveolus are being destroyed on all sides of the roots.

This destructive disease has formerly been the subject of considerable discussion by the profession and some success has been claimed in the treatment of it—even to the reproduction of the alveolar processes. But for the most part, dentists have abandoned the hope of success in restoring the lost tissue, or even checking the disease in such measure as to restore loose teeth to firmness in the jaw for any considerable length of time. So far as I have had experience, or have made observation of the treatment of others, the most that is accomplished, is, to render such teeth comfortable in the mouth, while the destruction goes on, though retarded by treatment.

Recently the attention of the profession has again been called to this disease by the supposed discovery of new pathological forms, and a correspondingly new treatment has been proposed. Dr. Riggs, of Boston, has been prominent in suggesting this new pathological condition, and for this reason we see it sometimes denominated "Rigg's Disease." His opinion is that, though primarily pertaining to the gum, in its wasting form, it pertains to the alveolar periosteum, which being destroyed by inflammatory action, necrosis and wasting of the parietes of the alveolus is certain to follow.

Whether this be the correct theory of the disease or not, is yet to be proved. So many sound and valuable teeth are annually lost through its ravages, it becomes the profession to entertain with favor, any suggestion that looks plausible as to diagnosis and treatment. Some physiological facts in relation to tooth development may help us in our judgment of the truth or falsity of this theory. When first formed the alveolus is a bony crypt completely walling in the growing tooth. As the period of the emergence of the tooth arrives the outer wall of the alveolus is absorbed to let the imprisoned tooth through. The crown having thus reached a full exposure the margin of the alveolus is reproduced to embrace the neck of the tooth. Through the prior sacular stage of development there is also a series of movements in emargination and reproduction of the alveoli in accommodation to the necessities of the forming teeth.

These changing movements are produced by the slightest exciting causes. It is sometimes assumed that there must be great pressure to induce absorption of bone. The growing tooth, however, but touches lightly if at all the inner wall of the alveolus, and at its gentlest touch the door is opened.

Now consider what this susceptibility to absorption and reproduction under the slightest physiological causes must become when all the tendencies are reversed, and natural excitants have been supplanted by destructive irritants. In this pathological state very slight causes are sufficient as irritants, to work destruction of the alveoli. It seems therefore reasonable to expect that the disease which begins in a *slight irritation of the gum* might, if continued, make destructive work, when it reaches the periosteum and the alveolar processes.

The treatment prepared, therefore, in accord with this theory is to remove by a surgical operation the necrosed and suppurating margins of the alveoli, and scrape and smooth the part operated upon down to the healthy bone.

This treatment is so in harmony with my own experience for years in the treatment of diseased gums, that I shall not hesitate to adopt it.

In a case which I have examined, and which will be presented to me for treatment immediately on my return to my office, I shall not only remove, as my custom is, the granulated and tumified gum which covers the wasting margins of the alveolus, but extend the operation with knife and caustics to the alveolar seat of the disease, and interestedly await results.—*Dental Register.*

